# Meta-analysis evaluating the impact of chili-pepper intake on all-cause and cardiovascular mortality: A systematic review

**DOI:** 10.1016/j.amsu.2021.102774

**Published:** 2021-09-08

**Authors:** Naser Yamani, Adeena Musheer, Priyanka Gosain, Saba Sarfraz, Humera Qamar, Muhammad Maaz Waseem, Muhammad Sameer Arshad, Talal Almas, Vincent Figueredo

**Affiliations:** a Department of Medicine, John H Stroger Jr. Hospital of Cook County, Chicago, IL, USA; b Department of Medicine, Dow University of Health Sciences, Karachi, Pakistan; c Department of Medicine, Memorial Healthcare System, Pembroke, Pines, USA; d Department of Medicine, Islamabad Medical and Dental College, Islamabad, Pakistan; e Department of Medicine, Khaja Bandanawaz Institute of Medical Sciences Gulbarga, India; f Department of Medicine, Baqai Medical University, Karachi, Pakistan; g Department of Medicine, RCSI University of Medicine and Health Sciences, Dublin, Ireland; h Department of Cardiology, St.Mary Medical Center, Langhorne, PA, USA

**Keywords:** Chili pepper, Mortality, Cardiovascular mortality, Cerebrovascular accident deaths, Cancer related mortality

## Abstract

**Background:**

Dietetics today occupy a significant place in the field of research, helping to discover cardiovascular benefits of healthy diets and consumption of organic foods such as fruits, vegetables, legumes, nuts, and whole grains. One of the components of vegetable-based diet is chili pepper (CP) which has been found to affect all-cause mortality.

**Methods:**

MEDLINE, EMBASE, Scopus, EBSCO, and Cochrane (Wiley) Central Register of Controlled Trials were searched from inception till January 9, 2020, identifying all relevant studies using keywords and truncations. Studies were included if (1) they were observational or randomized in nature (2) included patients consuming CP and (3) evaluated direct comparison between regular and rarely/never CP consumption.

**Results:**

Our preliminary search yielded 6976 articles. Post exclusion and after full-text screening, four potential observational studies with a population of 570,762. Pooled analysis found reduced all-cause mortality in CP consumers compared to nonconsumers with a risk ratio (RR) of 0.75 [95% CI: 0.64–0.88; p = 0.0004; I 2 = 97%]. The RR for CVD, cancer related and CVA deaths were 0.74 [95% CI: 0.62–0.88; p = 0.0006, I 2 = 66%], 0.77 [95% CI: 0.71–0.84; p = 0.0001; I 2 = 49%] and 0.76 [95% CI: 0.36–1.60; p = 0.47; I2 = 93%], respectively.

**Conclusion:**

Statistically significant results of our analysis put forward a rationale indicating an association between lower risk of all-cause, cardiovascular and cancer related deaths and CP consumption.

## Highlights

• Cardiovascular diseases have been observed to be a major cause of mortality.
• Recent transition to vegetable-based diet including Chili Pepper is positively impacting health of cardiovascular patients.
• Capsaicin affects TRPV1 Receptors and consequently weight-reduction.

## 1 Introduction

Dietetics today occupy a significant place in the field of research, helping to discover cardiovascular benefits of healthy diets and consumption of organic foods such as fruits, vegetables, legumes, nuts, and whole grains. One of the components of vegetable-based diet is chili pepper (CP) which has been found to affect all-cause mortality [1]. The chemical constituent of CP, capsaicin, has been shown to reduce all-cause mortality and deaths caused by CVD (cardiovascular disease), cancer and CVA (cerebrovascular accidents). However, absence of randomization and insufficient evidence [2] in previous studies has hindered demonstrating an association between CP consumption and mortality. This warrants a meta-analysis to study CP effects and benefits.

## 2 Methods

MEDLINE, EMBASE, Scopus, EBSCO, and Cochrane (Wiley) Central Register of Controlled Trials were searched from inception till January 9, 2020, identifying all relevant studies using keywords and truncations. Studies were included if (1) they were observational or randomized in nature (2) included patients consuming CP and (3) evaluated direct comparison between regular and rarely/never CP consumption. Primary outcome of interest was all-cause mortality and secondary outcomes included deaths by CVD, CVA and cancer. Pooled risk ratios and 95% confidence intervals were calculated using random-effect and generic inverse variance methods. A p-value <0.05 was considered significant. Reporting quality was evaluated using Preferred Reporting Items for Systematic Reviews and Meta-Analyses (PRISMA) [3] and methodological quality using the Assessment of Multiple Systematic Reviews (**AMSTAR**-**2**) tool [4].

## 3 Results

**Fig. 1:**
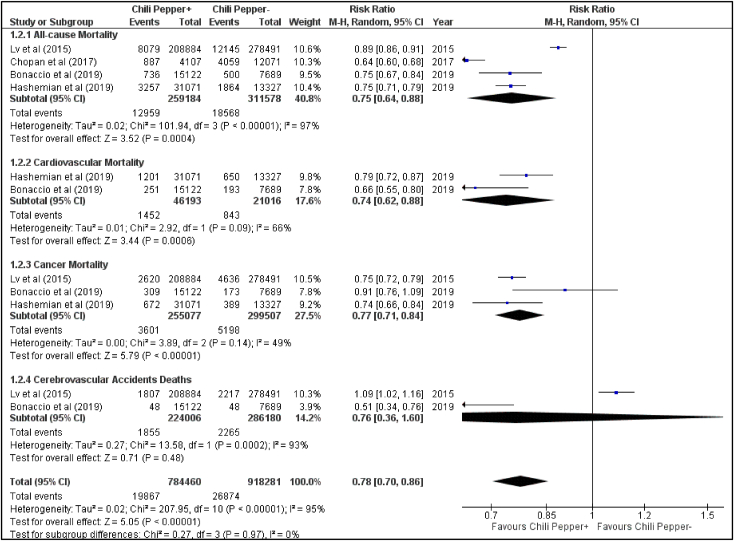
Forest plot displaying the effect of chili pepper consumption on all-cause mortality, cardiovascular mortality, cancer mortality, and cerebrovascular accident deaths using risk ratios (CI: Confidence Interval; M–H: Mantel-Haenzel).

Our preliminary search yielded 6976 articles. Post exclusion and after full-text screening, four potential observational studies with a population of 570,762 (259,184 consumed CP; 311,578 rarely/never consumed CP) met the inclusion criteria and thus included in the meta-analysis [1,[5], [6], [7]]. The studies used Food Frequency Questionnaire (FFQ), National Health and Nutrition Examination Survey (NHANES) to study the effects of CP consumption. Study characteristics are summarized in Table 1. Pooled analysis found reduced all-cause mortality in CP consumers compared to non-consumers with a risk ratio (RR) of 0.75 [95% CI: 0.64–0.88; p = 0.0004; *I*2 = 97%]. The RR for CVD, cancer related and CVA deaths were 0.74 [95% CI: 0.62–0.88; p = 0.0006, *I*2 = 66%], 0.77 [95% CI: 0.71–0.84; p = 0.0001; *I*2 = 49%] and 0.76 [95% CI: 0.36–1.60; p = 0.47; *I2* = 93%], respectively (Fig. 1).

**Table 1:** Characteristics of the studies included in the meta-analysis.

Study	Country	Years of enroll-ment	Type of study	Participants	Type of pepper	Intervention vs Control group (based on frequency of CP consumption)	Outcome Data Assessment	Ethnic Background	Food questionnaire	Follow-up (median in years)	Potential bias (adjustment)
Bonaccio et al. (2019)	Italy	2005 to 2010	Prospective cohort study; non-randomized	Men and women≥35 years of age	Chili pepper	CP consumers (n = 15122): up to 2 times/week to >4 times/week Rare/Non-consumers (n = 7689)	Italian mortality registry. Other outcome data were collected from medical records using ICD-9 coding	Moli-Sani, a southern Medi- terranean region in Italy	European Prospective Investigation into Cancer Food Frequency Questionnaire	8.2	Information/recall bias (confirmation of outcomes data with medical records). Possibility of residual and unobserved confounding
Hashemian et al. (2019)	Iran	2004 to 2008	Prospective cohort study; non-randomized	Individuals 40–75 years of age	Black or chili pepper	CP consumers (n = 31071): ever consumer of CP Non-consumers (n = 13327)	Death certificate and two internists evaluating the cause of death. Cause-specific mortality from the medical records using ICD-10 codes	Turkmen, non-Turkmen	116-item Food Frequency Questionnaire (FFQ)	11.1	At risk of selection bias
Chopan et al. (2017)	USA	1988 to 1994	Prospective cohort study; non-randomized	Adults ≥18 years including Mexican-American, other Hispanic, or non-Hispanic subjects	Hot red chili pepper	CP consumers (n = 4107): once per month or more Non-consumers (n = 12071)	Matching with National Death Index. Cause specific mortality was collected from medical records using ICD-10 codes	Multi-culture (White, Black, Hispanics)	81-item Food Frequency Questionnaire	18.9	Information/recall bias (extensive interviews)
Lv et al. (2015)	China	2004 to 2008	Prospective cohort study; non-randomized	10 geographically diverse areas across China, aged 30–79 years	Various types: fresh chili pepper, dried chili pepper, chili sauce, chili oil	CP consumers (n = 208884): At least once a week Rare/Non-consumers (n = 278491)	Linkage with death registries and residential records. Cause-specific mortality was collected using ICD*-10 codes	Chinese	Food Questionnaire: frequency of chili pepper intake (Never or almost never, only occasionally, 1 or 2 days a week, 3–5 days a week, or 6 or 7 days a week)	7.2	Residual confounding (inverse association between spicy food and mortality toward the null); At risk of selection bias

*International Classification of Diseases.

## 4 Discussion

This is the first meta-analysis carried out to assess the impact of CP consumption on all-cause, CVD and cancer related mortality. Our results show significant benefit from CP consumption in preventing such deaths as opposed to rare or no CP consumption. The lack of data on mode, quantity and frequency of CP consumption leads to non-standardization, along with variable populations in control and intervention groups leading to high heterogeneity level. The significant reduction of relative risk is supported by two potential processes. First, capsaicin promotes the activation of the TRPV1 (Transient receptor potential cation channel sub-family V member 1) receptor which through a cascade effect leads to thermogenesis, fat metabolism and other energy dissipation processes [8]. This way energy equilibrium shifts help in weight-reduction, consequently lowering the risk of CVD incidence [9]. Likewise, weight-reduction was observed in 30 participants in the study by Yoshioka et al. [10] where a diet rich in fat was supplemented with capsaicin. Second, theTRPV1, receptor found in epicardium, has been proposed to prevent myocardial infarction, through the release of substance P [11]. The TRPV1 dependent release of serotonin helps thrombin in platelet activation. This mechanism accounts for the pro-coagulating property of capsaicin and justifies the negative impact of CP on CVD and CVA deaths [12].

## 5 Conclusion

To our knowledge, this is the first systematic review and meta-analysis that attempt to identify association between CP consumption and mortality. Statistically significant results of our analysis put forward a rationale indicating an association between lower risk of all-cause, cardiovascular and cancer related deaths and CP consumption (Fig. 1).

## Declaration of competing interest

None to declare.

## Registration of research studies

1. Name of the registry: NA.

2. Unique Identifying number or registration ID: NA.

3. Hyperlink to your specific registration (must be publicly accessible and will be checked): NA.
