# Supplementary material for: Meta-analysis evaluating the impact of chili-pepper intake on all-cause and cardiovascular mortality: A systematic review
Source: Ann Med Surg (Lond). 2021 Sep 8;70:102774. doi: 10.1016/j.amsu.2021.102774 (PMC8463741; doi:10.1016/j.amsu.2021.102774)
Supplement: Multimedia component 3 [file mmc3.docx]

**AMSTAR 2 CHECK LIST**

**Box 1 AMSTAR 2 critical domains**

- $\surd$ Research questions and inclusion criteria for the review included the components of PICO (item 1)
- $\surd$ Review authors used a comprehensive literature search strategy (item 4)
- $\surd$ Review authors performed study selection in duplicate (item 5)
- $\surd$ Review authors performed data extraction in duplicate (item 6)
- $\surd$ Review authors described the included studies in adequate detail (item 8)
- $\surd$ Review authors reported any potential sources of conflict of interest, including any funding they received for conducting the review (item 16)

#### Box 2 Rating overall confidence in the results of the review

- $\surd$**High**
- No or one non-critical weakness: the systematic review provides an accurate and comprehensive summary of the results of the available studies that address the question of interest
- **Moderate**
- More than one non-critical weakness*: the systematic review has more than one weakness but no critical flaws. It may provide an accurate summary of the results of the available studies that were included in the review
- **Low**
- One critical flaw with or without non-critical weaknesses: the review has a critical flaw and may not provide an accurate and comprehensive summary of the available studies that address the question of interest
- **Critically low**
- More than one critical flaw with or without non-critical weaknesses: the review has more than one critical flaw and should not be relied on to provide an accurate and comprehensive summary of the available studies
- *Multiple non-critical weaknesses may diminish confidence in the review and it may be appropriate to move the overall appraisal down from moderate to low confidence
